# Impact of Skin Biopsy and Clinical-Pathologic Correlation in Dermatology Inpatient Consults

**DOI:** 10.7759/cureus.28534

**Published:** 2022-08-29

**Authors:** Amy Wells, Allison Harmel, Kristin N Smith, Paula Beers, Yingjie Qiu, Susmita Datta, Jennifer J Schoch, Anna De Benedetto, Isabel Longo, Kiran Motaparthi

**Affiliations:** 1 Department of Dermatology, Geisinger Commonwealth School of Medicine, Danville, USA; 2 Department of Ophthalmology, University of North Carolina School of Medicine, Chapel Hill, USA; 3 College of Medicine, University of Florida College of Medicine, Gainesville, USA; 4 Department of Dermatology, Malcolm Randall Veterans Affairs Medical Center, Gainesville, USA; 5 Department of Biostatistics and Health Data Science, Indiana University, Indianapolis, USA; 6 Department of Biostatistics, University of Florida College of Public Health and Health Professions, Gainesville, USA; 7 Department of Dermatology, University of Florida College of Medicine, Gainesville, USA; 8 Department of Dermatology, University of Rochester Medical Center, Rochester, USA; 9 Department of Dermatology, University of Florida, Gainesville, USA

**Keywords:** dermatology consultation, complex medical dermatology, biopsy, consult service, inpatient dermatology

## Abstract

Background

While studies of hospital dermatology have demonstrated diagnostic discordance between primary teams and dermatology consultants, little is known about the impact of biopsy and clinical-pathologic correlation (CPC) in consultation. This study compares biopsy performance based on diagnostic discordance and evaluates the impact of CPC on the diagnosis.

Methods

This was a retrospective review of 376 dermatologic consultations at a single academic medical center between July 1, 2017, and June 27, 2018.

Results

Biopsy was significantly less likely to be performed when the diagnosis by the referring primary team was unspecified (p < 0.001). In 24 percent of cases, the diagnosis based on histopathology alone differed from the diagnosis reached by formal CPC consensus review with either potential or significant impact on management.

Conclusion

Dermatologists who perform inpatient consultations and rely on hospital-based pathology services may consider a consensus review for CPC. Requests to perform a biopsy may be interpreted as a request for diagnostic assistance rather than pressure to perform a procedure.

## Introduction

Inpatient skin conditions represent a significant financial burden to the healthcare system, costing over 5 billion dollars annually and affecting one in eight hospitalized adults [1]. Given this burden, the role of dermatology services in the inpatient setting is expanding, with inpatient dermatology emerging as a unique subspecialty [2]. Inpatient dermatology consultation has been associated with improved patient outcomes, including reduced hospitalization length and a 10-fold reduction in the odds of readmission for patients with chronic skin conditions [3].

The primary reasons for dermatology consultation are to diagnose skin conditions and to perform biopsies [4,5]. While skin biopsy is an important tool in the evaluation of inpatient dermatologic conditions, the impact of biopsies on inpatient outcomes has yet to be addressed. In addition to highlighting the demographics of inpatients who receive dermatology consultation in our institution, we evaluated the frequency of biopsy performance based on the discordance between primary team clinical impression and dermatologist clinical impression. Additionally, we compared both dermatologist clinical impression and histopathology final impression to formal clinical-pathologic correlation (CPC) diagnosis to determine the impact of consensus review on diagnosis in inpatient dermatology.

## Materials and methods

Data collection

We conducted a retrospective review of all dermatology inpatient consults at a large academic medical center from July 1, 2017 to June 27, 2018. Data was obtained using a list of medical record numbers from all inpatient dermatology consultation requests. Variables collected included age, sex, race, biopsy performance, and primary team, dermatologist, and dermatopathologist diagnosis. The primary team requesting consultation provided their presumptive diagnoses at the time of the request. Glass slides, clinical images, and patient histories from all biopsies performed were reviewed weekly in a CPC consensus meeting consisting of dermatologists who perform inpatient consultation and hospital-based, pathology-trained dermatopathologists.

Discordances between dermatologist versus primary team diagnosis, histopathologic versus CPC consensus diagnosis, and dermatologist versus CPC consensus diagnosis were rated by two dermatologists who were not involved in the consults. Discordance ratings were classified into one of four categories: N, A, B, or C. “Type N” reflected no discordance. “Type A” reflected discordance without impact on management, for example, lichen simplex chronicus versus prurigo nodularis. “Type B” reflected discordance with a potential impact on management, for example, morbilliform drug eruption versus acute generalized exanthematous pustulosis. “Type C” reflected discordance with a significant impact on management, for example, cellulitis versus urticaria. For the purpose of the study, diagnostic concordance included type N and type A categories, while diagnostic discordance included type B and C categories.

The final clinical diagnoses were subdivided into 12 categories: papulosquamous disorders, infections, vesiculobullous disorders, adnexal disorders, rheumatologic diseases, neoplasms, vascular diseases, genodermatoses, disorders of subcutaneous fat, metabolic diseases, urticarias, erythemas, purpura, and other. The category “other” included psychogenic diseases and unspecified diagnoses such as “non-specific rash”. These categories included the final diagnosis regardless of biopsy status.

Statistical analysis

Numerical variables were summarized by means ± standard deviations (SD) (range). Categorical variables were summarized by frequencies (proportions) and compared between groups using a Chi-square (χ2) test; the binary independent variable was the agreement between diagnosis and the ordinal dependent variable was the impact on the treatment. P-values were 2-sided and considered significant at the 0.05 level. R (3.6.1) was used for the analysis.

## Results

The characteristics of the patient population are listed in Table 1. There were 376 patients included in the study, with 48 percent male and 52 percent female. The average age of patients was 42 ± 27 years for those who received a biopsy and 50 ± 22 years for those who did not receive a biopsy. In 87.2 percent of consults, the primary team specified a diagnosis. Biopsy was performed in 44.7 percent of cases.

**Table 1:** Demographic and clinical characteristics of patients who received dermatology inpatient consultation. Mean ± Standard Deviation {min-max}; No.: number

	No biopsy performed	Biopsy performed	p-value
Number of patients	208	168	
Gender, No. (%)			
Male	99 (48)	80 (48)	
Female	109 (52)	88 (52)	
Age	42 ± 26 {0 – 97}	50 ± 22 {1-95}	
Race, No. (%)			
African American	48 (23)	38 (23)	
Asian	2 (1)	2 (1)	
Asian Indian	1 (0)	2 (1)	
Caucasian	137 (66)	114 (68)	
Greek	1 (0)	0 (0)	
Hispanic	14 (7)	9 (5)	
Latina	1 (0)	0 (0)	
Latino	1 (0)	0 (0)	
Native American	0 (0)	1 (1)	
Unknown	3 (1)	2 (1)	
Readmission, No. (%)	95 (46)	84 (50)	0.465
Deceased, No. (%)	34 (16)	NA	
Dermatology-related readmission, No. (%)			
Yes	12 (13)	19 (23)	0.102
No	84 (88)	64 (77)	
Length of stay (admission for all diagnoses)	16 ± 27 {0-270}	17 ± 36 {0-383}	0.969
Admission for dermatologic condition, No. (%)			
Yes	57 (27)	80 (48)	<0.001
No	151 (73)	87 (52)	

Comparison of biopsy performance based on concordance between primary team clinical impression and dermatologist clinical impression

Skin biopsy was significantly more likely to be performed only in cases of type B discordance (potential clinical impact, p = 0.025). Skin biopsy was not more likely to be performed for concordant diagnoses or type C discordances (p = 0.422 and p = 0.710, respectively). Of note, a biopsy was significantly less likely to be performed when the diagnosis by the referring primary team was unspecified (p < 0.001, Table 2).

**Table 2:** Biopsy performance based on discordance of clinical diagnosis between primary team and dermatologists. No.: number

	No biopsy performed	Biopsy performed	P-value
Concordance, No. (%)	65 (31)	60 (36)	0.422
Unspecified, No. (%)	40 (19)	8 (5)	<0.001
Type B discordance, No. (%)	14 (7)	24 (14)	0.025
Type C discordance, No. (%)	89 (43)	76 (45)	0.71
Overall discordance, No. (%)	103 (50)	100 (59)	0.067

Comparison of dermatologist clinical impression to consensus CPC diagnosis

The rate of concordance between initial dermatologist clinical impression and final CPC diagnosis was 85 percent. Compared to CPC consensus, dermatologist diagnosis was discordant in 15 percent of cases, with type B and type C discordance of 4 and 11 percent respectively.

Comparison of histopathology impression to consensus CPC diagnosis

When comparing histopathologic impression to diagnosis based on formal consensus review, reliance on histopathology alone would produce an overall discordance of 24 percent. Type B and type C discordance would account for 7 and 18 percent, respectively.

Comparison of concordance between primary team clinical impression and dermatologist clinical impression by final diagnosis category

Among the subdivided clinical diagnoses, infections had the highest rate of discordance (75%) between the hospitalist and dermatologist clinical impressions (Table 3). This category most commonly included cellulitis, herpesvirus infections, scabies, and tinea. Of the discordances within the infectious category, 97% reflected type C discordance (data not shown). The diagnostic category of urticarias, erythemas, and purpura was associated with a discordance rate of 61%, which was mostly due to drug eruptions and vasculitis. Rheumatologic diseases were associated with a discordance rate of 65%; however, this diagnostic group only included 17 diagnoses, most of which were dermatomyositis and lupus.

**Table 3:** Concordance between primary team clinical impression and dermatologist clinical impression by diagnostic category. No.: number

	Discordance No. (%)	Concordance No. (%)
Urticarias, erythemas, and purpura	65 (61)	42 (39)
Infections	66 (75)	22 (25)
Papulosquamous disorders	15 (54)	13 (46)
Rheumatologic diseases	11 (65)	6 (35)
Vascular diseases	6 (38)	10 (63)
Neoplasms	7 (47)	8 (53)
Adnexal disorders	6 (55)	5 (45)
Vesiculobullous disorders	4 (44)	5 (56)
Disorders of subcutaneous fat	1 (20)	4 (80)
Metabolic diseases	4 (80)	1 (20)
Genodermatoses	2 (50)	2 (50)
Other	5 (9)	48 (91)

## Discussion

In this study, biopsy was performed in 46 percent of inpatients who received a dermatology consultation; in prior studies, this rate ranged from 29 to 40 percent [6,7,8]. Diagnostic discordance between primary teams and dermatologists ranges between 22 to 52 percent [9], with changes to treatment in 58 to 96 percent of cases [5-7,10-13]. Our study demonstrated an overall diagnostic discordance of 54 percent between primary teams and dermatologists. A significantly impactful discordance in diagnosis or an unspecified diagnosis by the primary team was not associated with a greater likelihood of biopsy. Diagnostic discordance between dermatology and primary team diagnoses may partially result from the lack of in-depth exposure to dermatology in medical school and primary care training [12]. However, a study evaluating a five-year dermatology lecture series specific for primary teams failed to improve the diagnostic concordance rate between primary teams and dermatologists, underlining the importance of expert consultation [14]. Our study also highlights the high rates of discordance between hospitalists and dermatologists that affects infectious diagnoses, usually resulting in a significant change in patient management. While some cutaneous infections can be managed appropriately by nondermatologists, these data highlight the importance of dermatologist consultation in the diagnosis of skin infections in the hospital setting. Taken together with the results of this study, while up to 31 percent of dermatology consultations specifically request biopsy [4], this may be interpreted as a request for diagnostic assistance rather than pressure to perform a procedure.

Consensus review for CPC was impactful in diagnosis and management for up to one in four patients. Therefore, dermatologists who perform inpatient consultations and rely on hospital-based pathology services may consider a formal consensus review for CPC. When compared to the consensus diagnosis based on CPC, the leading clinical impression by consulting dermatologists was correct in 85 percent of cases. This high percentage of appropriate clinical diagnosis prior to biopsy highlights the utility of inpatient dermatology.

There are several limitations to this study. First, all skin conditions were analyzed together. An investigation of each category of skin disease may reveal a more nuanced impact of biopsy on treatment plans and outcomes. Additionally, this review relied on data from requested dermatology consults instead of all dermatology-related inpatient encounters. In cases where consultation was not requested, it is unclear if dermatology consultation and/or biopsy would have changed management. Informal communication between dermatologists and pathologists prior to consensus review for severe or urgent diagnoses may have confounded results made at consensus review. Finally, these findings reflect a retrospective study at a single academic medical center with a dedicated inpatient dermatology service and formalized consensus conference for CPC; these features may limit generalizability to other medical centers. We recognize that many medical centers lack an inpatient dermatology service. This study demonstrates the importance of following up with an outpatient dermatologist rather than a nondermatologist in those settings.

## Conclusions

In summary, our results demonstrate that the decision to perform a skin biopsy during an inpatient dermatology consultation should rely on the clinical impression of the dermatologist. Additionally, consensus review for CPC is impactful and may be considered for inpatient dermatology services that rely on hospital-based pathology. Finally, primary teams should be encouraged to request a consultation and rely on inpatient dermatologists to provide guidance on the need for a skin biopsy.
